# Attitudes of Jordanian Medical Students Toward People With Mental Illness

**DOI:** 10.7759/cureus.84982

**Published:** 2025-05-28

**Authors:** Osama A Zitoun, Rayan M Joudeh, Adnan R Alnaser, Abdelkader Battah, Dana W Al-Mansour, Yazan Faraj, Anas Y El-Massad, Abdulhaleem Elshaikh Khalil, Mahmoud Kashoua, Robert Rosenheck

**Affiliations:** 1 School of Public Health Sciences, University of Waterloo, Ontario, CAN; 2 Faculty of Medicine, Ibn Sina University for Medical Sciences, Amman, JOR; 3 Geoffrey Jefferson Brain Research Centre, Northern Care Alliance NHS Foundation Trust, Manchester, GBR; 4 Department of Laboratory and Anatomy, Jordan University, Amman, JOR; 5 School of Medicine, Jordan University of Science and Technology, Irbid, JOR; 6 School of Medicine, V. N. Karazin Kharkiv National University, Kharkiv, UKR; 7 School of Medicine, Sulaiman Alrajhi University, Qassim, SAU; 8 Department of Psychiatry, Yale School of Medicine, New Haven, USA

**Keywords:** attitude, jordan, medical students, mental illness, stigma

## Abstract

Background

For people with mental illnesses, the stigma in the healthcare system and among healthcare providers has been identified as a major barrier to accessing treatment and achieving recovery, as well as poor physical treatment. This nationwide cross-sectional study assessed stigma toward mental illnesses among medical students in Jordan. This study aimed to assess the prevalence and patterns of stigma toward mental illnesses among medical students in Jordan and examine the influence of psychiatric education and specialty preferences on stigma levels.

Methodology

A nationwide cross-sectional study was conducted between April 21 and April 30, 2021, targeting medical students from all six medical schools in Jordan. Participants completed a validated, structured, electronically distributed questionnaire assessing attitudes toward mental illness. Exploratory factor analysis (EFA) identified three key domains, i.e., (1) Social Acceptance, (2) Policies to Promote Community Integration, and (3) Beliefs about Treatability of Mental Illness. Demographic information included age, academic year (pre-clinical vs. clinical), and psychiatric training exposure (classroom and/or clinical). Data analysis was performed using SPSS (IBM Corp., Armonk, NY, USA). A total of 2,104 medical students participated. The age distribution was as follows: 24.8% were aged 18-19.9 years, 43.6% were aged 20-22.9 years, and 31.7% were aged ≥23 years. Participants included both pre-clinical and clinical-year students from diverse academic institutions across Jordan.

Results

Clinical-year students reported significantly higher (less stigmatizing) scores in all three domains compared to pre-clinical students (p < 0.001). Those exposed to both classroom and clinical psychiatry training showed more favorable attitudes than those with limited or no exposure. Students interested in psychiatry specialization exhibited lower (more stigmatizing) scores in the treatability and community integration domains compared to peers interested in surgical (p < 0.001, p = 0.003) and medical specialties (p < 0.001, p = 0.001).

Conclusions

While a majority of students expressed social acceptance and supported community integration of individuals with mental illness, stigma persisted in perceptions of treatability. Greater exposure to psychiatric education correlates with reduced stigma. Unexpectedly, students inclined toward psychiatry showed more stigmatizing views in some domains, warranting further investigation. These findings highlight the importance of enhancing mental health education across the medical curriculum.

## Introduction

Stigma is a deeply discrediting trait that has the power to transform the view toward an individual from an ordinary person into a devalued person [1]. Stigma can be defined as interrelated knowledge problems (ignorance), attitude problems (bias), and behavior problems (discrimination) [2]. Stigma is “a mark of disgrace associated with a particular circumstance, quality, or person, which leads to prejudice and discrimination” [3]. Studies suggest that the general public regards those who suffer from mental illness as frightening, strange, and capable of performing unpredictable acts [4]. Therefore, the risk of getting hurt is often considered an important aspect in the development of stigma. Embarrassing physical appearance, devaluing labels, and psychological symptoms can be considered socially regarded stigmata for patients suffering from psychiatric illness [5]. Society often believes that behavioral and mental disorders can be personally controlled, and if individuals cannot improve on their own, they are seen to lack personal motivation [6] and viewed as personally responsible for their condition.

For people with mental illnesses, the stigma in the healthcare system and among healthcare providers has been identified as a major barrier to accessing treatment and recovery, as well as poor physical health. It also impacts help-seeking from healthcare providers and becomes a negative factor in their work environment [7]. A 2024 study reported that approximately 26.1% of Jordan’s population suffers from mental disorders, placing the country on the higher end of the global prevalence range [8]. Jordanians have the same negative thoughts toward patients with mental illness as in other countries [9]. A good number of these negative thoughts are built on false beliefs about mental illness. For instance, the belief that a person might acquire mental illness from evil spirits or as a result of witchcraft or the evil eye [10]. According to folk beliefs in the Jordanian community, transfer of a “negative” force can cause the development of mental illness [10]. El-Islam et al. reported that such cultural beliefs are deeply ingrained and not easily erased by modern medical education [11].

The attitudes of medical students toward people with mental illness have drawn increasing scholarly attention over the past few years [12]. Numerous studies have attempted to assess the attitudes of medical students toward people with mental illness and the factors that influence these attitudes, such as gender, age, familiarity with mental illnesses, presence of family members with mental illness, knowledge about mental illness, and the interaction with impacted patients [13]. The effects of gender on stigmatization attitudes are mixed. A study from Papua New Guinea [14] reported no effect of gender on stigmatization attitudes. On the other hand, a study conducted among the general population of Finland reported that males express a higher level of stigma toward mental illnesses compared to females [15]. In Jordan, one study found females to have less stigmatizing attitudes than males [16]. One of these studies was conducted among pre-clinical and post-clinical medical students and graduate psychiatrists. Other studies targeting both the general population [14] and university students [15] have emphasized the positive effect of a family history of mental illness on reducing stigma among students with a previous history of receiving mental health services [14,15].

In Jordan, the subject of mental health stigma has been largely underexplored, with only a few published studies addressing the issue. One study surveying 519 university students reported low scores on the Attitudes Toward Seeking Professional Psychological Help, reflecting a general reluctance to seek psychological support [14]. Complementing these findings, a study found that many Jordanian nursing students attributed mental illness to supernatural causes such as jinn, magic, or the evil eye, as well as personal weakness [17]. These culturally rooted beliefs not only contribute to stigma but also significantly reduce the likelihood of help-seeking behaviors. This clear contrast underscores the urgent need to strengthen mental health education in Jordan to counteract misconceptions and reduce stigma. Nevertheless, levels of stigma toward psychiatric illness are not only high among the general population but also among healthcare professionals [18]. Most studies aiming to assess stigma among medical students focus more on students in clinical years than those in pre-clinical years. It is unclear whether attitudes toward mental disorders are influenced by medical education. This study aims to assess the attitudes of medical students and interns toward mental illness to determine whether the following factors are associated with stigma: the level of medical education, exposure to psychiatry classes and clerkships, students’ interest in specific specialties after graduation, personal experience with mental illness, and other demographic characteristics.

## Materials and methods

Study design and setting

This study employed an online, survey-based, cross-sectional design, given COVID-19-related restrictions at the time of data collection (April 21-30, 2021). The study was conducted across six accredited Jordanian medical schools: the University of Jordan, Jordan University of Science & Technology, Hashemite University, Mutah University, Al-Balqa Applied University, and Yarmouk University. Medical interns, including those who graduated abroad but were interning in Jordan, were also included.

Sampling method

A convenience sampling approach was used. Approximately 10,000 students were estimated to be enrolled across the six schools. All currently enrolled medical students and medical interns in Jordan were invited to participate. The following inclusion criteria were considered for inclusion in the study: (1) currently enrolled as a medical student or medical intern in Jordan, (2) provided informed consent, and (3) completed the full survey. The following exclusion criteria were considered for exclusion from the study: (1) not currently enrolled students or interns, (2) declined consent, or (3) incomplete responses.

Study groups

Students were asked to report their level of exposure to psychiatry education. Based on their responses, they were categorized into the following three groups: Group 1: no psychiatry education (n = 804); Group 2: classroom psychiatry education only (n = 545); and Group 3: classroom and clerkship psychiatry education (n = 755). These groupings reflect the structure of medical education in Jordan, where psychiatry is taught through a classroom-based course in the third year and a clinical clerkship in the fifth year.

Data collection

Approval was obtained from the deans of all six medical schools. The questionnaire was distributed via students’ official university emails and through social media channels by student representatives. The survey included an introductory page explaining the aim and scope of the study, the voluntary nature of participation, the anonymity of responses, and participants’ right to withdraw at any time. Students had to provide informed consent electronically before proceeding with the questionnaire. Data collection occurred from April 21 to April 30, 2021.

Assessment tools

The questionnaire had the following three parts: demographics, a comprehensive stigma survey, and the Patient Health Questionnaire-4 (PHQ-4). PHQ-4 is a validated and reliable four-item screening tool for anxiety and depression. It consists of two items, each for anxiety and depression subscales [19]. A score of 3 or more on any subscale indicates possible anxiety or depression. The stigma survey, the main component assessing attitudes toward mental illness, was adapted from validated scales, i.e., the Fear and Behavioral Intentions Toward the Mentally Ill [20], the Community Attitudes Toward the Mentally Ill [21], and the WPA Schizophrenia Stigma Scale [22]. This combined survey has been used internationally in Brazil, Nigeria, the United States, Saudi Arabia, the Philippines, and China [23-26]. Internal consistency was evaluated via exploratory factor analysis, and only items with a loading ≥0.4 and a Cronbach’s alpha >0.7 were retained.

Data analysis

Data were extracted into Microsoft Excel (Microsoft Corp., Redmond, WA, USA) and then analyzed using SPSS Statistics (IBM Corp., Armonk, NY, USA). Negative stigma items were recoded into positive anti-stigmatizing statements for consistency. Exploratory factor analysis was conducted on the 48-item stigma scale to identify coherent factors. Demographic variables, psychological distress (via PHQ-4), and experience with mental illness were analyzed. Statistical tests, including analysis of variance (ANOVA), chi-square, and t-tests, assessed differences in stigma factors across demographic and educational groups. A significance level of α < 0.05 was used.

Ethical considerations

The study adhered to the Declaration of Helsinki and was approved by the Institutional Review Board (IRB) at the University of Jordan (meeting number: 7/2021; reference number: 101/2021/9341). Participants provided informed consent electronically before completing the survey.

## Results

Attitudes toward mental illness

This study surveyed a total of 2,104 students from six medical schools in Jordan. Exploratory factor analysis with orthogonal (varimax) rotation using principal axis factoring highlighted three distinctive factors reflecting attitudes and beliefs toward mental illness. Factor 1 (Social Acceptance) consisted of 12 items with a Cronbach’s alpha of 0.79, Factor 2 (Policies to Promote Community Integration) consisted of seven items with a Cronbach’s alpha of 0.75, and Factor 3 (Treatability of Mental and Medical Illnesses) consisted of six items with a Cronbach’s alpha of 0.79 (Tables 1, 2).

**Table 1:** Exploratory factor analysis of student’ attitudes toward mental illness. Factor loadings represent correlations between survey items and extracted factors. Only items with a loading ≥0.4 are shown.

Factors	Item loading		
	Factor 1	Factor 2	Factor 3
Factor 1 – Social Acceptance			
People with mental illness are not a public nuisance	0.503		
People with mental illness are not dangerous because of violent behavior	0.509		
I would not be afraid to have a conversation with a person who has a mental illness	0.52		
I would not be upset or disturbed about working on the same job	0.408		
I would be able to maintain a friendship	0.4		
I would be willing to share a room	0.403		
I am not afraid of people with mental illness	0.575		
I would invite somebody into my home if I knew him/her suffered from mental illness	0.451		
It is not frightening to think of people with mental problems living in residential neighborhoods	0.477		
Anyone with a mental illness could be given any responsibility	0.436		
Anyone with a history of mental problems should not be excluded from taking public office	0.508		
I would accept living next door to someone who has been mentally ill	0.434		
Factor 2 – Policies to Promote Community Integration			
We have a responsibility to provide the best possible care for people with mental illness		0.543	
We need to adopt a far more tolerant attitude toward people with mental illness in our society		0.593	
As far as possible, mental health services should be provided through community-based facilities		0.56	
People with mental illness are far less of a danger than most people suppose		0.483	
If somebody had been a former psychiatric patient, I could have them as a friend		0.481	
If somebody who had been a former patient came to live next door to me, I would greet them occasionally		0.531	
I would have casual conversations with neighbors who had suffered from mental illness		0.419	
Factor 3 – Treatability of Mental and Medical Illnesses			
How treatable is schizophrenia			0.575
How treatable is depression			0.645
How treatable is bipolar disorder			0.668
How treatable is anxiety			0.626
How treatable is hypertension			0.54
How treatable is diabetes mellitus			0.548

**Table 2:** Descriptive statistics of the identified factors from the questionnaire. Cronbach’s alpha was used to assess internal consistency (α > 0.7). Statistical significance threshold was set at p-values <0.05.

	Factor 1 – Social Acceptance	Factor 2 – Policies to Promote Community Integration	Factor 3 – Treatability of Mental and Medical Illnesses
Number of items	12	7	6
Mean	0.722	0.92	2.073
Standard error	0.00514	0.00373	0.01459
Cronbach’s alpha	0.79	0.75	0.79

Sociodemographic factors and their association with stigma factors

Demographic details are displayed in Table 3. The mean age of the students was 21.3 years (range = 17-32 years). As numerous age variables were reported, age was organized into the following three categories: 18-19.9, which included 24.8% of students (n = 521); 20-22.9 years, which included 43.6% of students (n=917), and ≥23 years, which included 31.7% of students (n = 666). Female students constituted nearly three-fifths of the students (n = 1,234, 58.7%). They scored significantly higher than males in relation to the mean score of Factor 2 (p < 0.001). Students’ level of study was organized into the following three categories: basic pre-clinical years (first to third year of study), which included 52.4% of students (n = 1,103); clinical years (fourth to sixth year of study), which included 38.5% of student (n = 810); and interns, which included 9.1% of students (n = 191). Both clinical-year students and interns scored higher than basic pre-clinical-year students in the mean score of Factor 1 (p < 0.001) and Factor 2 (p < 0.001). Clinical-year students also scored higher than pre-clinical-year students in the mean score of Factor 3 (p < 0.001). Interns who had graduated from a medical school outside Jordan constituted 2.9% of the students (n = 62) and scored higher than interns who had graduated from Jordanian medical schools on the mean score of Factor 1 (0.86 vs. 0.73; p < 0.001). More than one-fourth (n = 568, 27.0%) of the students reported personal experience with mental illness, and one-half (n = 1,071, 50.9%) had experience through family or friends. Self-experience with mental illness was significantly associated with a higher mean score in Factor 1 (p <0.001), while experience with mental illness through family or friend was associated with a higher score on Factor 1 (p < 0.001), Factor 2 (p = 0.001), and Factor 3 (p < 0.001). The majority of the students screened positive for anxiety and depression (n = 1,488, 70.7% and n = 1,475, 70.1%, respectively), as indicated by the PHQ-4 scale. Screening positive for anxiety was associated with a higher mean score in Factor 2 (p =0.035) and Factor 3 (p =0.006).

**Table 3:** Demographic characteristics and its associated factors reflecting attitudes toward mental illness. Mean differences assessed using analysis of variance or t-tests as appropriate. Statistical significance is defined as p-values <0.05. *: First to third year of medical school; **: fourth to fifth year of medical school. PHQ-4 = Patient Health Questionnaire-4

Demographic characteristics		Count	Percent	Factor 1 – Social Acceptance		Factor 2 – Policies to Promote Community Integration		Factor 3 – Treatability of Mental and Medical Illnesses	
				Mean	P-value	Mean	P-value	Mean	P-value
Overall		2,104	100%	0.72	-	0.92	-	2.10	-
Age (years)	18–19.9	521	24.8%	0.69	<0.001	0.88	<0.001	1.92	<0.001
	20–22.9	917	43.6%	0.71		0.92		2.12	
	≥23	666	31.7%	0.76		0.94		2.13	
Gender	Male	870	41.3%	0.71	0.16	0.90	<0.001	2.04	0.057
	Female	1,234	58.7%	0.73		0.93		2.10	
Study level	Basic years^*^	1,103	52.4%	0.69	<0.001	0.90	<0.001	2.01	<0.001
	Clinical years^**^	810	38.5%	0.75		0.93		2.16	
	Internship	191	9.1%	0.78		0.955		2.10	
Place of graduation	Still a medical student	1,911	90.8%	0.72	<0.001	0.92	0.009	2.07	0.421
	Inside Jordan	131	6.2%	0.73		0.95		2.13	
	Outside Jordan	62	2.9%	0.86		0.96		2.01	
Self-experience with mental illness	No	1,536	73.0%	0.71	<0.001	0.92	0.89	2.08	0.652
	Yes	568	27.0%	0.75		0.92		2.06	
Family/Friend experience with mental illness	No	1,033	49.1%	0.68	<0.001	0.91	0.001	2.02	<0.001
	Yes	1,071	50.9%	0.76		0.93		2.13	
PHQ-4 subscale to screen for anxiety	Normal	616	29.3%	0.72	0.826	0.91	0.035	2.01	0.006
	Screened positive for anxiety	1,488	70.7%	0.72		0.92		2.10	
PHQ-4 subscale to screen for depression	Normal	629	29.9%	0.73	0.123	0.91	0.148	2.10	0.438
	Screened positive for depression	1,475	70.1%	0.72		0.92		2.10	

Psychiatric education and attitudes toward mental illness

Regarding psychiatric education, Group 1 students, who had no prior psychiatric education, constituted 38.2% (n = 804) of the study population, Group 2 students, who had completed the psychiatry classroom, course constituted slightly more than one-fourth of the study population (n = 545, 25.9%), and Group 3 students, who had completed both psychiatry classroom course and clinical clerkship, constituted 35.9% of the study population (n = 755) (Table 4). ANOVA and subsequent independent t-tests showed that Group 3 students scored significantly higher than students in both Groups 2 and 1 in the mean scores of Factor 1 (0.76 vs. 0.73 vs. 0.68; p < 0.001) and Factor 2 (0.95 vs. 0.92 vs. 0.90; p < 0.001). Further, Group 3 students scored better in the mean score of Factor 3 than those in Group 1 (2.14 vs. 1.95; p < 0.001). Similarly, Group 2 students scored better in the mean score of Factor 3 than those in Group 1 (2.16 vs. 1.95; p < 0.001), with no significant difference between Groups 2 and 3 concerning Factor 3, as shown in Table 5.

**Table 4:** Comparison of medical students’ attitudes by their psychiatry education level. Analysis of variance is used to compare each group with factors. Statistical significance is defined as p-values <0.05.

	Group 1 (no psychiatry education), n = 804	Group 2 (classroom psychiatry education), n = 545	Group 3 (classroom and clerkship psychiatry education), n = 755			
	Mean score (SE)	Mean score (SE)	Mean score (SE)	F	P-value	Paired comparisons
Factor 1	0.68 (0.0085)	0.73 (0.0098)	0.76 (0.0082)	27.7	<0.001	3 > 2 > 1
Factor 2	0.90 (0.0072)	0.92 (0.0069)	0.95 (0.0048)	16.1	<0.001	3 > 2 > 1
Factor 3	1.95 (0.026)	2.16 (0.026)	2.14 (0.023)	22.1	<0.001	3 > 1; 2 > 1

**Table 5:** Comparison of mean scores of factors according to students’ psychiatry education level. Independent sample t-tests is used to compare the factor mean among the grouping assigned. Statistical significance is defined as p-values <0.05.

	Mean (SE) of the first group	Mean (SE) of the second group	t-value	P-value
Factor 1 paired comparisons				
Group 3 vs. Group 2	0.76 (0.0082)	0.73 (0.0098)	2.34	0.02
Group 3 vs. Group 1	0.76 (0.0082)	0.68 (0.0085)	7.32	<0.001
Group 2 vs. Group 1	0.73 (0.0098)	0.68 (0.0085)	4.31	<0.001
Factor 2 paired comparisons				
Group 3 vs. Group 2	0.95 (0.0048)	0.92 (0.0069)	3.38	0.001
Group 3 vs. Group 1	0.95 (0.0048)	0.90 (0.0072)	5.65	<0.001
Group 2 vs. Group 1	0.92 (0.0069)	0.90 (0.0072)	2.03	0.043
Factor 3 paired comparisons				
Group 3 vs. Group 2	2.14 (0.023)	2.16 (0.026)	-0.42	0.673
Group 3 vs. Group 1	2.14 (0.023)	1.95 (0.026)	5.59	<0.001
Group 2 vs. Group 1	2.16 (0.026)	1.95 (0.026)	5.6	<0.001

Planned specialty and attitudes toward mental illness

Table 6 displays the association between the students’ planned specialty and their attitudes, which are referred to by the mean scores of Factors 1, 2, and 3. Nearly one-half of the study students (n = 1,044, 49.62%) planned to pursue surgical specialties, such as General Surgery, Orthopedics, Otorhinolaryngology, Neurosurgery, Cardiothoracic Surgery, Obstetrics and Gynecology, and Emergency Medicine after graduation. Around 39.12% (n = 823) planned to pursue medical specialties, such as Internal Medicine, Family Medicine, Pediatrics, Cardiology, Oncology, Radiology, Geriatrics, Nephrology, Endocrinology, and Gastroenterology. Only 8.98% (n = 189) were interested in Psychiatry, and 2.28% (n = 48) had no plans to specialize after graduation. Students interested in psychiatry scored significantly lower in relation to the mean score of Factor 2 than those interested in surgical specialties (0.855 vs. 0.932, p < 0.001) and medical specialties (0.855 vs. 0.920, p < 0.001). Again, students who chose psychiatry as their planned specialty had significantly lower scores in Factor 3 when compared to those who chose surgical specialties (1.91 vs. 2.07, p = 0.003), medical specialties (1.91 vs. 2.11, p = 0.001), and those who had no plan to specialize after medical school (1.91 vs. 2.16, p = 0.035) (Table 7).

**Table 6:** Comparison of medical students’ attitudes by planned specialty. Analysis of variance was used to compare factor mean scores with students’ planned specialty. Statistical significance is defined as p-values <0.05. ^a^: General Surgery, Orthopedics, Otorhinolaryngology, Neurosurgery, Cardiothoracic Surgery, Obstetrics and Gynecology, Emergency, etc.; ^b^: Internal Medicine, Family Medicine, Pediatrics, Cardiology, Oncology, Radiology, Geriatrics, Nephrology, Endocrinology, Gastroenterology, etc.

	Surgical specialties^a^	Medical specialties^b^	Psychiatry	No plans to specialize			
	(n = 1,044)	(n = 823)	(n = 189)	(n = 48)			
	Mean score (SE)	Mean score (SE)	Mean score (SE)	Mean score (SE)	F	P-value	Paired comparisons
Factor 1	0.712 (0.0072)	0.738 (0.0082)	0.705 (0.019)	0.724 (0.033)	2.32	0.073	-
Factor 2	0.932 (0.0046)	0.920 (0.0064)	0.855 (0.015)	0.911 (0.029)	11	<0.001	1 > 3; 2 > 3
Factor 3	2.07 (0.0205)	2.11 (0.023)	1.91 (0.056)	2.16 (0.092)	5.08	0.002	1 > 3; 2 > 3; 4 > 3

**Table 7:** Comparing factor mean scores according to students’ planned specialty. Independent sample t-tests used to assess the association between the factors and students’ planned specialty. Statistical significance is defined as p-values <0.05. ^a^: General Surgery, Orthopedics, Otorhinolaryngology, Neurosurgery, Cardiothoracic Surgery, Obstetrics and Gynecology, Emergency, etc; ^b^: Internal Medicine, Family Medicine, Pediatrics, Cardiology, Oncology, Radiology, Geriatrics, Nephrology, Endocrinology, Gastroenterology, etc.

	Mean (SE) of the first group	Mean (SE) of the second group	t-value	P-value
Factor 2 pair comparisons				
Surgical vs. Medical	0.932 (0.0046)	0.920 (0.0064)	1.56	0.119
Surgical vs. Psychiatry	0.932 (0.0046)	0.855 (0.015)	4.77	<0.001
Surgical vs. No plans	0.932 (0.0046)	0.911 (0.029)	0.72	0.475
Medical vs. Psychiatry	0.920 (0.0064)	0.855 (0.015)	3.87	<0.001
Medical vs. No plans	0.920 (0.0064)	0.911 (0.029)	0.33	0.744
Psychiatry vs. No plans	0.855 (0.015)	0.911 (0.029)	-1.64	0.102
Factor 3 pair comparisons				
Surgical vs. Medical	2.07 (0.0205)	2.11 (0.023)	-1.394	0.163
Surgical vs. Psychiatry	2.07 (0.0205)	1.91 (0.056)	2.99	0.003
Surgical vs. No plans	2.07 (0.0205)	2.16 (0.092)	-0.972	0.331
Medical vs. Psychiatry	2.11 (0.023)	1.91 (0.056)	3.36	0.001
Medical vs. No plans	2.11 (0.023)	2.16 (0.092)	-0.543	0.587
Psychiatry vs. No plans	1.91 (0.056)	2.16 (0.092)	-2.19	0.035
Surgical vs. Medical	2.07 (0.0205)	2.11 (0.023)	-1.394	0.163

## Discussion

This study assessed attitudes toward people with mental illness among medical students in Jordan. It also examined how these attitudes varied by psychiatric education, age, gender, academic level, graduation origin, personal experience, and psychological distress. Factor analysis identified the following three key dimensions: Factor 1: Social Acceptance, Factor 2: Community Integration Policies, and Factor 3:Treatability of Mental and Medical Illnesses. The study targeted around 10,000 medical students in Jordan. In total, 2,104 students participated in the study from all six medical schools in Jordan, along with international graduates working as interns in Jordanian hospitals. When compared with the male gender, the female gender was associated with significantly better mean scores regarding policies to promote community integration (Factor 2). This could be further explained by the level of sympathy that females exhibit. This finding is consistent with the results of other studies conducted in Finland [14] and another in Jordan [15].

Findings on the influence of gender on stigmatizing attitudes toward mental illness are quite mixed. A study from Papua New Guinea reported no effect of gender on stigmatization attitudes [13], while a study conducted among the general population in Finland found that males expressed higher levels of stigma toward mental illness compared to females [14]. Similarly, a study conducted in Jordan found that females held less stigmatizing attitudes than males [15]. In addition to gender, other factors such as older age and lack of familiarity with mental illness have also been associated with more negative attitudes [14]. Interestingly, a study from Portugal involving healthcare professionals reported significantly lower stigma levels among psychiatrists compared to physicians in other specialties and medical students [16]. Furthermore, the previously mentioned study from Papua New Guinea also found that students in their final year of study who had completed their psychiatry rotation demonstrated lower levels of stigma compared to those who had not yet received psychiatric training [15].

Regarding students’ level of study, clinical-year students and interns displayed better attitudes regarding both Factor 1 and Factor 2 than basic pre-clinical-year students, which can be explained by the greater amount of knowledge and exposure to mental illness among senior students. In contrast, a study from the Philippines found that students with higher levels of training displayed more negative attitudes. The researchers attributed this to the fact that graduate physicians often encounter patients with severe mental health issues in hospital psychiatric wards, where they face challenging behaviors, including violence, which underscores the harsh realities of severe mental illness [25].

Moreover, psychiatric education influenced students’ attitudes. Completion of both the psychiatry clerkship and classroom course was significantly associated with higher mean scores in Factors 1 and 2 than completion of the classroom course only, which was also significantly better than having no psychiatry education at all (p < 0.001). Students who attended psychiatry clerkship achieved a significant improvement in their knowledge and interest in psychiatry, which, in turn, enhanced their acceptance of mental illnesses and reduced stigmatizing attitudes compared to students who had not yet encountered psychiatric patients or those who had only completed the theoretical component of psychiatry [26], highlighting the value of clinical training and encountering patients with mental illness. The contact theory, which suggests that interactions with individuals with mental illness improve attitudes and acceptance toward mental illness [27], supports this finding. Furthermore, students who had attended the classroom course with or without the clinical clerkship reported higher mean scores in their beliefs regarding the treatability of mental illnesses (Factor 3). This finding can also be explained by the effect of psychiatric education on medical students in terms of knowledge regarding the treatability of mental illnesses, in contrast with basic pre-clinical-year students with no exposure to psychiatry, who rated it negatively.

Interestingly, interns who attended medical studies outside Jordan showed better anti-stigmatizing attitudes, scoring significantly higher in Factor 1, than those trained locally. This may reflect differences in the emphasis placed on psychiatry in international medical curricula, as well as the influence of acculturation, where exposure to more mental health-inclusive cultures could foster greater acceptance [26]. This may indicate that low resourcing of mental care services and scarcity of psychiatric hospitals in developing countries are negatively influencing stigmatizing attitudes. Furthermore, while a previous study from Saudi Arabia reported no difference in attitudes and beliefs among those interested in surgical specialties, medical specialties, and psychiatry [24], another study from Italy reported that students who preferred medical specialties over surgical ones appeared to show fewer stigmatizing attitudes toward people with mental disorders [28]. Surprisingly, we found that students interested in the psychiatry specialty scored significantly lower in mean scores related to policies to promote community integration (Factor 2) and treatability of mental illnesses (Factor 3) than those interested in major surgical and medical specialties. It might seem surprising, as a student interested in pursuing a career in psychiatry would be expected to have a better understanding and exposure, which enhances their attitudes and exposure and improves their beliefs and attitudes towards mental illness. However, it seems that having this exposure in low-resource mental health centers within a developing country like Jordan might expose them more to poorly treated people with severe mental disorders. Partially similar findings were noted in the previously mentioned study that reported higher stigmatization attitudes among medical students in Ghana when compared to those in Australia and another study from Bangladesh that reported more stigmatizing attitudes among senior medical students who were exposed to people with mental disorders within low-funded hospitals struggling with a shortage of specialized mental health resources [26].

Those who had personal experience with mental illness reported better social acceptance than those who reported no such experience, while those who had personal experience through their friends and families scored higher not only regarding social acceptance but also in their beliefs about promoting community integration and treatability of mental illnesses than those who did not. Similarly, Ando et al. found a positive influence of personal experiences and direct contact with people with mental illness [29]. While having depressive symptoms did not significantly affect students’ attitudes, screening positive for anxiety was associated with a slightly more anti-stigmatizing higher mean score in Factor 2 and Factor 3, which could be attributed to personal experience. The alarmingly high rates of medical students screening positive for both anxiety and depression, around 70% for both, should also be taken into consideration. Nevertheless, these rates could be explained by known stressors among medical students [30]. The data were collected during the COVID-19 pandemic, during which psychological distress was significantly exacerbated among students globally, which could have contributed to these elevated rates among medical students in Jordan.

This study identified clear intergroup differences in stigma attitudes. Female students showed more positive attitudes toward community integration, consistent with findings from Finland and Jordan [14,15], though some studies, such as from Papua New Guinea, found no gender effect [13]. Clinical-year students and those with psychiatric training scored higher in social acceptance and integration, supporting contact theory [26], while contrasting findings from the Philippines suggest negative experiences with severely ill patients may worsen attitudes [24]. Interns trained abroad scored higher in social acceptance, possibly due to better psychiatric education or acculturation [25]. Interestingly, students interested in psychiatry showed more negative attitudes, perhaps due to exposure to under-resourced mental health settings in Jordan. Finally, personal or indirect experience with mental illness, as well as screening positive for anxiety, was linked to more favorable attitudes, echoing past research [28].

One limitation of this study is the timing of data collection during the peak of the second wave of the COVID-19 pandemic in April 2021. This may have influenced students’ attitudes toward mental illness and contributed to the elevated rates of psychological distress. To account for this, we included the PHQ-4 scale to assess anxiety and depression levels, allowing some interpretation of the students’ mental state and its potential impact on their responses. Additionally, the cross-sectional design limits the ability to infer causal relationships between exposure variables and attitudes. The use of self-reported questionnaires may introduce social desirability and recall biases, and the online nature of the survey may have affected response accuracy or introduced sampling bias, particularly among students with limited internet access or motivation to complete the survey. These factors may limit the generalizability of the findings. Regarding strengths, our study is the first of its type in Jordan with a large number of students. The study sample is representative of the population of medical students in Jordan; however, there is a lack of data to compare the sample with those who did not complete the assessment. Most studies aiming to assess stigma among medical students focus more on students in clinical years and less on those in pre-clinical years. It is unclear whether several attitudes about mental disorders are influenced during distinct levels of medical education [29]. Students from all levels of medical education were included to gain a comprehensive view. This study demonstrated the effect of a classroom psychiatry course and a four-week psychiatry clinical training on medical students’ attitudes. Moreover, we commented on the effect of personal experience with mental illness, mental state, and planned medical specialties after graduation on the attitudes of medical students toward mental illness.

These findings have important implications for medical schools in Jordan, particularly regarding curriculum design and the integration of psychiatric education. There is a need to strengthen psychiatric training at both undergraduate and postgraduate levels, including the development of mandatory courses on psychiatric disorders and their treatment for medical graduates. Notably, the finding that students interested in psychiatry exhibited more negative attitudes toward the treatability of mental illness and policies promoting community integration warrants further investigation. We recommend conducting qualitative studies to explore these attitudes in depth and better understand the underlying factors shaping perceptions of mental illness among medical students in Jordan.

## Conclusions

This study highlights the importance of psychiatric education in shaping medical students’ attitudes toward mental illness. The findings suggest a need for curricular reforms that integrate psychiatry more meaningfully throughout medical training. Enhancing exposure to both theoretical and clinical aspects of mental health may foster more positive, evidence-based attitudes. Additionally, the unexpected finding of more negative beliefs among students interested in psychiatry underscores the complexity of stigma and calls for deeper investigation. Future research, including qualitative studies, is warranted to explore the underlying factors influencing students’ perceptions and to inform targeted interventions in medical education, particularly in developing countries like Jordan.

## Appendices

**Table 8:** Survey on medical students’ attitudes and beliefs about mental illness.

Demographics	
Age ________ in years	
Gender	Male
	Female
Current study level	First-year medical student
	Second-year medical student
	Third-year medical student
	Fourth-year medical student
	Fifth-year medical student
	Sixth-year medical student
	Internship/Graduate
University	University of Jordan
	Yarmouk University
	Mutah University
	Al-Balqa Applied University
	Hashemite University
	Jordan University of Science and Technology
	Other (graduated from a university outside Jordan and doing an internship)
For medical interns: Where did you complete your MBBS undergraduate studies?	Inside Jordan
	Outside Jordan
Psychiatry Education	
Have you completed your psychiatry classroom course?	Yes, I have completed it (or are currently taking it)
	No, have not taken it (or not applicable)
Have you completed your psychiatry clinical clerkship/rotation?	Yes, I have completed it (or are currently taking it)
	No, have not taken it yet
Total duration of psychiatry-related education/training (formal, year 1 to year 6)?	Zero
	Less than 1 month
	1–2 months
	2–3 months
	3–4 months
	4–5 months
	More than +5 months
Have you taken any extra psychiatry training (summer courses, electives, webinars, etc.)?	Yes
	No
Planned specialty	
What is your planned specialty?	Psychiatry
	Medical specialties (e.g., Internal Medicine, Family Medicine, Pediatrics, Cardiology, Oncology, Radiology, Geriatrics, Nephrology, Endocrinology, Gastroenterology, etc.)
	Surgical specialties (e.g., General Surgery, Orthopedics, ENT, Neurosurgery, Cardiothoracic Surgery, Obstetrics and Gynecology, ER, etc.)
	None - No plans to specialize
Opinions on the treatability of illnesses	
In your opinion, how treatable are the following illnesses: schizophrenia	Not at all treatable
	Mostly not treatable
	Substantial improvement possible
	Can be cured to near normal
	Can be completely cured
Major depression	Not at all treatable
	Mostly not treatable
	Substantial improvement possible
	Can be cured to near normal
	Can be completely cured
Hypertension	Not at all treatable
	Mostly not treatable
	Substantial improvement possible
	Can be cured to near normal
	Can be completely cured
Bipolar disorder	Not at all treatable
	Mostly not treatable
	Substantial improvement possible
	Can be cured to near normal
	Can be completely cured
Anxiety disorder	Not at all treatable
	Mostly not treatable
	Substantial improvement possible
	Can be cured to near normal
	Can be completely cured
Diabetes	Not at all treatable
	Mostly not treatable
	Substantial improvement possible
	Can be cured to near normal
	Can be completely cured
Personal experience with mental illness	
Do you have personal experience with mental illness (yourself)?	Yes
	No
Do you have personal experience with mental illness (family or close friend)?	Yes
	No
Beliefs about the causes of mental illness	
Which of the following do you think can cause mental illness?	
Drug/alcohol misuse	Yes
	No
Possession by evil spirits	Yes
	No
Traumatic event/shock	Yes
	No
Stress	Yes
	No
Genetic inheritance	Yes
	No
Physical abuse	Yes
	No
Biological factors (non-brain/genetics)	Yes
	No
God’s punishment	Yes
	No
Witchcraft	Yes
	No
Brain disease	Yes
	No
Poverty	Yes
	No
Curse	Yes
	No
Beliefs about people with mental illness	
People with mental illness	
Can be treated outside a hospital	Yes
	No
Tend to be mentally retarded	Yes
	No
Can work in regular jobs	Yes
	No
Are a public nuisance	Yes
	No
Can work in regular jobs	Yes
	No
Are they dangerous due to violent behavior?	Yes
	No
Attitudes toward interacting with people with mental illness	
In interacting with someone with mental illness, would you:	
Be afraid to have a conversation	Yes
	No
Be upset about working together	Yes
	No
Maintain a friendship	Yes
	No
Refuse to share a room	Yes
	No
Feel ashamed if a family member has a mental illness	Yes
	No
Be willing to marry someone with a mental illness	Yes
	No
Social distance and stigma	
Are you afraid of people with mental illness?	Yes
	No
Would you object to having mentally ill people in your neighborhood?	Yes
	No
Would you avoid conversations with neighbors with mental illness?	Yes
	No
Would you work with someone with a mental illness?	Yes
	No
Would you invite someone with a mental illness to your home?	Yes
	No
Would you have a former psychiatric patient as a friend?	Yes
	No
Would you greet a former psychiatric patient neighbor?	Yes
	No
Would you have casual conversations with such neighbors?	Yes
	No
Would you visit a former psychiatric patient neighbor?	Yes
	No
Social and policy beliefs	
Does locating mental health facilities downgrade the neighborhood?	Agree
	Disagree
Is it frightening to think of mentally ill people in residential neighborhoods?	Agree
	Disagree
Would you prefer not to live next to someone who has a mental illness?	Agree
	Disagree
Is it foolish for a woman to marry a man who has a mental illness, even if recovered?	Agree
	Disagree
Should people with mental illness be given responsibility?	Agree
	Disagree
Should people with a history of mental problems be excluded from public office?	Agree
	Disagree
Are people with mental illness a burden on society?	Agree
	Disagree
Should people showing signs of mental disturbance be hospitalized immediately?	Agree
	Disagree
Should we provide the best possible care for people with mental illness?	Agree
	Disagree
Can virtually anyone become mentally ill?	Agree
	Disagree
Is increasing mental health spending a waste of money?	Agree
	Disagree
Do people with mental illness deserve sympathy?	Agree
	Disagree
Should society adopt a more tolerant attitude?	Agree
	Disagree
Have people with mental illness been ridiculed for too long?	Agree
	Disagree
Should mental health services be community-based?	Agree
	Disagree
Are people with mental illness less dangerous than believed?	Agree
	Disagree
Should less emphasis be placed on protecting the public from them?	Agree
	Disagree
Is community integration the best therapy?	Agree
	Disagree
Should residents fear mental health patients accessing neighborhood services?	Agree
	Disagree
Should people with mental illness have equal job rights?	Agree
	Disagree
Can former female psychiatric patients be trusted as babysitters?	Agree
	Disagree
Is mental illness like any other illness?	Agree
	Disagree
Should no one be excluded from neighborhoods due to mental illness?	Agree
	Disagree
Are mental hospitals outdated?	Agree
	Disagree
Are people with mental illness more violent?	Agree
	Disagree
PHQ-4 screening (past 14 days)	
Feeling nervous, anxious, or on edge	Not at all
	Several days
	More than half the days
	Nearly every day
Unable to stop or control worrying	Not at all
	Several days
	More than half the days
	Nearly every day
Feeling down, depressed, or hopeless	Not at all
	Several days
	More than half the days
	Nearly every day
Little interest or pleasure in doing things	Not at all
	Several days
	More than half the days
	Nearly every day
